# Brain Network Modularity Predicts Improvements in Cognitive and Scholastic Performance in Children Involved in a Physical Activity Intervention

**DOI:** 10.3389/fnhum.2020.00346

**Published:** 2020-09-03

**Authors:** Laura Chaddock-Heyman, Timothy B. Weng, Caitlin Kienzler, Robert Weisshappel, Eric S. Drollette, Lauren B. Raine, Daniel R. Westfall, Shih-Chun Kao, Pauline Baniqued, Darla M. Castelli, Charles H. Hillman, Arthur F. Kramer

**Affiliations:** ^1^Beckman Institute, The University of Illinois at Urbana-Champaign, Urbana, IL, United States; ^2^Department of Diagnostic Medicine, The University of Texas at Austin, Austin, TX, United States; ^3^Department of Psychology, University of Colorado, Denver, CO, United States; ^4^Department of Kinesiology, The University of North Carolina at Greensboro, Greensboro, NC, United States; ^5^Department of Psychology, Northeastern University, Boston, MA, United States; ^6^Helen Wills Neuroscience Institute, University of California, Berkeley, Berkeley, CA, United States; ^7^Brain and Creativity Institute, University of Southern California, Los Angeles, CA, United States; ^8^Department of Kinesiology and Health Education, The University of Texas at Austin, Austin, TX, United States; ^9^Department of Physical Therapy, Movement, and Rehabilitation Sciences, Northeastern University, Boston, MA, United States

**Keywords:** academic achievement, brain networks, brain network modularity, children, cognition, physical activity, scholastic performance

## Abstract

**Introduction**: Brain network modularity is a principle that quantifies the degree to which functional brain networks are divided into subnetworks. Higher modularity reflects a greater number of within-module connections and fewer connections between modules, and a highly modular brain is often interpreted as a brain that contains highly specialized brain networks with less integration between networks. Recent work in younger and older adults has demonstrated that individual differences in brain network modularity at baseline can predict improvements in performance after cognitive and physical interventions. The use of brain network modularity as a predictor of training outcomes has not yet been examined in children.

**Method**: In the present study, we examined the relationship between baseline brain network modularity and changes (post-intervention performance minus pre-intervention performance) in cognitive and academic performance in 8- to 9-year-old children who participated in an after-school physical activity intervention for 9 months (*N* = 78) as well as in children in a wait-list control group (*N* = 72).

**Results**: In children involved in the after-school physical activity intervention, higher modularity of brain networks at baseline predicted greater improvements in cognitive performance for tasks of executive function, cognitive efficiency, and mathematics achievement. There were no associations between baseline brain network modularity and performance changes in the wait-list control group.

**Discussion**: Our study has implications for biomarkers of cognitive plasticity in children. Understanding predictors of cognitive performance and academic progress during child development may facilitate the effectiveness of interventions aimed to improve cognitive and brain health.

## Introduction

Cognitive processes such as executive functions (inhibition, working memory, mental flexibility), attention, and memory are known to play a role in successful goal-directed behavior and scholastic performance (St. Clair-Thompson and Gathercole, 2006; Bull et al., 2008). School performance can predict success in later years (Kuncel et al., 2004; Kuncel and Hezlett, 2007), and academic placement, and educational program effectiveness, and school funding are often determined by children’s performance on standardized academic tests. Thus, it is important to determine biomarkers and correlates of academic progress as well as lifestyle factors that positively influence cognitive function and scholastic performance.

Scientists have developed interventions aimed to improve executive function and scholastic performance during childhood and across the lifespan. As the brain develops structurally and functionally during childhood, this period of neurodevelopment may be particularly sensitive to lifestyle factors and intervention. For example, participation in physical activity is a promising intervention to improve cognitive and brain health during childhood and across the lifespan (Hillman et al., 2014; Donnelly et al., 2016; Kramer and Colcombe, 2018; Chaddock-Heyman et al., 2019). In particular, participation in physical activity and higher levels of aerobic fitness is positively related to cognitive function, scholastic performance, and brain health in preadolescent children (for a review see Chaddock-Heyman et al., 2014). Physically active and higher fit children outperform less active and lower fit children on cognitive and scholastic tasks, and the performance differences are paralleled by differences in brain structure and brain function (for reviews see Chaddock-Heyman et al., 2014; Donnelly et al., 2016).

Recently, scientists have begun to examine whether baseline (pre-intervention) brain properties, such as properties of brain networks, can predict improvements in performance with physical and cognitive training interventions. Brain networks are said to exhibit a modular organization, such that they are comprised of modules or sub-networks. The brain can be segregated into network modules based on connectivity patterns among individual brain regions, or nodes. Network modules reflect groupings of nodes that share high connectivity among each other. Using a mathematical approach called graph theory, a modularity metric is calculated based on the degree of within-module connections compared to between-network connections (Newman and Girvan, 2004). Higher modularity reflects a greater number of within-module connections and fewer connections between modules. A highly modular brain can be interpreted as a brain that contains highly specialized brain networks with less integration between networks.

Individual differences in baseline brain network modularity, measured during a resting-state functional MRI scan, have been found to predict improvements (i.e., changes) in performance after cognitive and physical interventions (Arnemann et al., 2015; Gallen et al., 2016; Baniqued et al., 2018, 2019; for review see Gallen and D’Esposito, 2019). In one study (Gallen et al., 2016), healthy older adults with more modular brain networks at baseline showed greater improvements on tasks involving the synthesis of complex information after cognitive training, with no predictive power of modularity in a control group (Gallen et al., 2016). In addition, in young adults involved in cognitive training with casual video games that engaged reasoning and working memory processes, baseline network modularity was positively associated with training-related improvements on untrained tasks, with no associations in participants who did not show training gains (Baniqued et al., 2019). Similarly, in patients with traumatic brain injury (TBI), higher brain network modularity at baseline was associated with greater improvements on tasks of executive function after goal-oriented attention and self-regulation training (Arnemann et al., 2015). Finally, Baniqued et al. (2018) examined whether baseline brain network modularity predicted cognitive improvements after a physical activity intervention in healthy older adults. In older adults who showed gains in aerobic fitness and cognitive function, higher brain modularity at baseline predicted greater gains in executive function from pre-intervention to post-intervention (Baniqued et al., 2018). Together, these studies suggest that brain modularity may hold predictable power across populations and interventions aimed at enhancing cognition. In the four studies in older adults, younger adults, and TBI patients, individuals with a more modular brain network organization before training were more likely to benefit from cognitive or physical intervention.

To our knowledge, the use of brain network modularity as a predictor of training outcomes has not yet been examined in children. In the present study, we examined the relationship between baseline (pre-intervention) brain network modularity and changes (post-intervention minus pre-intervention) in cognitive and academic performance in 8- to 9-year-old children who participated in an after-school physical activity intervention for 9 months compared to children randomized to a wait-list control group. We hypothesized that children in the physical activity intervention with higher baseline modularity would show greater gains in cognitive and scholastic performance compared to those with lower modularity. That is, children in the intervention may be able to better capitalize on higher levels of brain modularity to derive greater benefit from physical activity intervention. We did not have any specific predictions about baseline brain network modularity and performance changes in the wait-list control group.

## Materials and Methods

Children were recruited from schools in East-Central Illinois. Eligible participants were required to: (1) be 7- to 9-years-old; (2) have an absence of school-related learning disabilities (i.e., individual education plan related to learning), adverse health conditions, physical incapacities, or neurological disorders; (3) qualify as prepubescent (Tanner pubertal timing score; Taylor et al., 2001); (4) report no use of medications that influence central nervous system function; (5) demonstrate right-handedness as measured by the Edinburgh Handedness Questionnaire (Oldfield, 1971); (6) complete a mock MRI session to screen for claustrophobia in an MRI machine; and (7) sign an informed assent approved by the Institutional Review Board of the University of Illinois at Urbana-Champaign. A legal guardian also provided written informed consent following the Institutional Review Board of the University of Illinois at Urbana-Champaign. The guardian was asked to provide information regarding participants’ socioeconomic status (SES), as determined by: (1) participation in the free or reduced-price lunch program at school; (2) the highest level of education obtained by the mother and father; and (3) number of parents who worked full-time (Birnbaum et al., 2002). Participants also completed the Woodcock–Johnson III paper-and-pencil test to assess intelligence quotient (IQ) and cognitive function (Woodcock, 1997).

The Institutional Review Board of the University of Illinois at Urbana-Champaign approved the present study. MRI scans were obtained at the Biomedical Imaging Center of the Beckman Institute of the University of Illinois, both pre-intervention and post-intervention (The post-intervention scans are not included in the present study). Children completed the cognitive tasks and scholastic performance assessment on a separate day, both pre-intervention and post-intervention, and testing occurred in a quiet, sound-attenuated room in a one-on-one setting. Children were compensated $15/h for MRI testing and $10/h for the neuropsychological testing.

**Figure 1:**
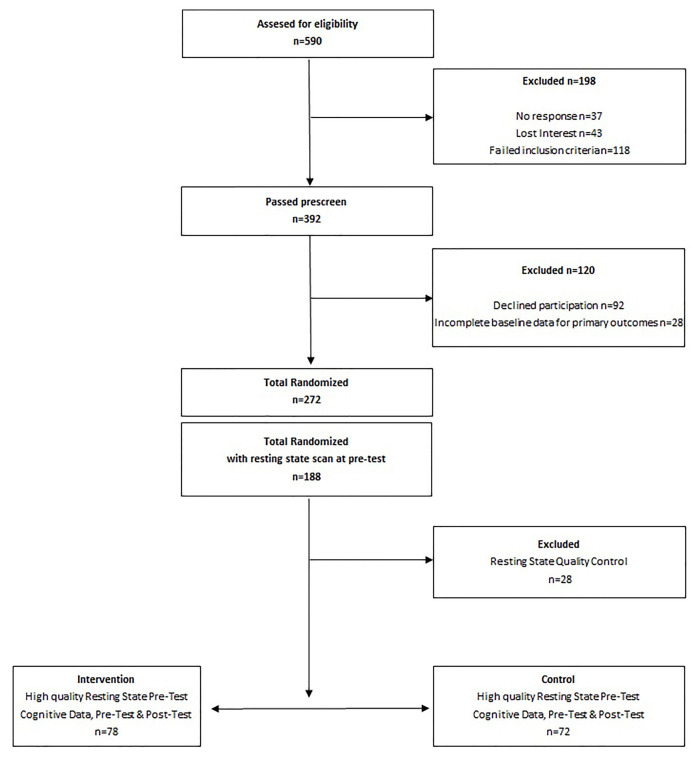
Study design.

Please see Figure 1 for an illustration of the study design. Five hundred ninety children were assessed for eligibility for the FITKids2 study, and 198 were excluded due to no response (*N* = 37), loss of interest (*N* = 43), or failure of the inclusion criteria (*N* = 118). Three hundred ninety-two children passed prescreening, 92 children declined participation and 28 children had incomplete baseline data for primary outcomes. Two-hundred and seventy-two children were randomized into the FITKids2 physical activity intervention, and 188 children completed the resting state MRI scan at baseline (pre-intervention). Twenty-eight children were excluded following quality control checks of functional scans. Functional scans were excluded if more than 20% of volumes exhibited framewise displacement (FD) above 0.2 mm or if mean relative motion was greater than 0.5 mm. Cognitive and modularity measures greater than or less than three standard deviations from the mean were also excluded (*N* = 2 outlier exclusions for baseline brain network modularity, *N* = 1 for Cognitive Efficiency, *N* = 2 for Thinking Ability, *N* = 3 for Verbal Ability, *N* = 1 for Reading; results remain the same when outlier data points were included in the sample).

The present study included a total of 150 children–78 children in the physical activity intervention (45 girls and 33 boys, mean age = 8.7 years, age range 7.8–9.9 years, grades 2–4) and 72 children in the wait-list control group (37 girls and 34 boys, mean age = 8.6 years, age range 7.9–9.9 years, grades 2–4). See Table 1 for participant information.

**Table 1:** Mean (SD) for physical activity and waitlist control groups at baseline (pre-intervention) and post-intervention.

	Physical activity		Control	
	Baseline	Post	Baseline	Post
Age (years)	8.7 (0.5)	–	8.6 (0.5)	–
Gender	45 girls, 33 boys	–	37 girls, 34 boys	–
IQ (General)	109.2 (15.4)	–	110.9 (13.0)	–
Pubertal timing	1.4 (0.5)	–	1.3 (0.4)	–
SES	1.9 (0.8)	–	1.9 (0.7)	–
VO_2__max_ (ml/kg/min)	42.4 (7.3)	42.5 (7.3)	43.0 (7.1)	43.0 (6.4)
VO_2__max_ percentile	35.8 (30.2)	35.5 (29.6)	38.0 (30.4)	37.5 (28.9)
Reading achievement	110.5 (13.9)	110.6 (14.0)	113.1 (14.4)	115.5 (16.0)
Mathematics achievement*	108.3 (16.2)	110.6 (17.9)	111.1 (16.2)	114.0 (17.0)
Executive processes (WJ)*	107.0 (10.4)	111.6 (9.8)	109.7 (9.8)	113.1 (9.9)
Thinking ability (WJ)	113.9 (12.9)	119.2 (12.4)	115.7 (12.8)	120.0 (13.6)
Cognitive efficiency (WJ)*	98.8 (16.7)	102.3 (17.8)	98.9 (15.5)	105.2 (16.1)
Verbal ability (WJ)	107.9 (12.4)	109.1 (12.6)	109.3 (11.5)	112.1 (11.5)
Modularity (6%)	0.488 (0.062)	–	0.474 (0.065)	–

Note: Woodcock–Johnson III paper and pencil tasks (Woodcock, 1997); SES—Socioeconomic Status (Low: < 2; Moderate: 2–3; High, >3). *p < 0.05. Association between baseline network modularity and change in performance in children involved in the physical activity intervention (uncorrected).

### Woodcock–Johnson Battery of Cognitive Tasks

Children completed subtests from the Woodcock–Johnson III Tests of Cognitive Abilities (WJ III; Woodcock, 1997). Individual cognitive tests were administered to participants, and combinations of the individual tests form clusters that represent general categories of broad cognitive abilities. The cognitive performance clusters include Executive Processes, Thinking Ability, Cognitive Efficiency, and Verbal Ability.

The cognitive cluster of Executive Processes includes tasks of cognitive flexibility and rule switching (Concept Formation), sequential reasoning and spatial scanning (Planning), and attention and interference control (Pair Cancelation). During the Concept Formation task, participants were asked to identify rules and concepts that created geometric shapes. The Concept Formation task provides a measure of cognitive flexibility, rule application, and rule switching. During the Planning task, participants were asked to trace unique shapes without retracing or picking up the pencil. The Planning task provides a measure of sequential reasoning, spatial scanning, and speed in visually surveying a spatial field. During the Pair Cancellation task, participants were asked to circle two target shapes when the shapes appeared in a sequence (for 3 min). The Pair Cancellation task measures attention, concentration, and interference control.

The cognitive cluster of Thinking Ability represents fluid reasoning, visual-spatial thinking, and processing of non-language information *via* short term memory (*via* performance on tasks of Visual Auditory Learning, Spatial Relations, Sound Blending, and Concept Formation). During the Visual Auditory Learning task, participants were orally presented words that were associated with visual symbols and then asked to translate the visual symbols. The Visual Auditory Learning task measures recall of verbal labels for visual symbols. During the Spatial Relations task, participants were asked to rotate a shape *via* imagination and/or select the components of shape. The Spatial Relations task measures the ability to visualize and adjust spatial shapes and forms. During the Sound Blending task, participants were asked to name a complete word after listening to the individual syllables and phonemes that form the word, thereby providing a measure of phonetic coding. The Thinking Ability cluster also includes task performance on the task of Concept Formation.

The cognitive cluster of Cognitive Efficiency represents perceptual speed, short term memory, and the ability to store and recode information (*via* performance on tasks of Visual Matching and Numbers Reversing). During the Visual Matching task, participants must quickly find and circle two identical numbers in a row of six numbers in 3 min, thereby providing a measure of perceptual speed. During the Numbers Reversed task, participants were asked to repeat a span of random numbers in reverse order, thus providing a measure of the ability to temporarily store and recode orally presented information.

The cognitive cluster of Verbal Ability is reflected by performance on a task of Verbal Comprehension, which consists of picture vocabulary, synonyms, antonyms, and verbal analogies.

### Scholastic Performance

The scholastic performance was assessed with subtests from the Kaufman Test of Educational Achievement, Second Edition (Kaufman and Kaufman, 2004). Standardized scores (Mean = 100, *SD* = 15) for reading (word recognition and reading comprehension) and mathematics (math concepts and applications and math computation) were determined. Kaufman Test of Educational Achievement, Second Edition subtests have very high internal consistencies, inter-rater reliabilities, and internal validity (*r* = 0.91–0.97).

Reading achievement was determined by performance on tasks of word recognition and reading comprehension. Specifically, the word recognition subtest involved pronouncing words of gradually increasing difficulty. The reading comprehension subtest involved reading words and pointing to the corresponding picture, acting out the action of words, and answering questions about reading passages.

Mathematics achievement was determined by performance on tasks of math concepts, math applications, and math computation. The math concepts and application subtest consisted of basic math concepts such as comparing numbers and rounding numbers, as well as problems requiring algebra, calculus, and trigonometry (88 items). The math computation subtest was a paper-and-pencil test involving the addition, subtraction, multiplication, and division of whole numbers and fractions (72 items).

### Aerobic Fitness Testing

Children completed a VO_2max_ test to assess aerobic fitness. The aerobic fitness of each child was measured as maximal oxygen consumption (VO_2max_) during a graded exercise test (GXT). The GXT employed a modified Balke Protocol and was administered on a LifeFitness 92T motor-driven treadmill (LifeFitness, Schiller Park, IL, USA) with expired gases analyzed using a TrueOne2400 Metabolic Measurement System (ParvoMedics, Sandy, Utah). Children walked and/or ran on a treadmill at a constant speed with increasing grade increments of 2.5% every 2 min until volitional exhaustion occurred.

Oxygen consumption was measured using a computerized indirect calorimetry system (ParvoMedics True Max 2400) with averages for VO_2_ and respiratory exchange ratio (RER) assessed every 20 s. A polar heart rate (HR) monitor (Polar WearLink + 31; Polar Electro, Finland) was used to measure HR throughout the test, and ratings of perceived exertion (RPE) were assessed every 2 min using the children’s OMNI scale (Utter et al., 2002). Maximal oxygen consumption was expressed in ml/kg/min and VO_2 max_ was based upon maximal effort as evidenced by: (1) a plateau in oxygen consumption corresponding to an increase of less than 2 ml/kg/min despite an increase in workload; (2) a peak HR ≥185 beats per minute (American College of Sports Medicine, 2006) and an HR plateau (Freedson and Goodman, 1993); (3) RER ≥1.0 (Bar-Or, 1983); and/or (4) a score on the children’s OMNI RPE scale ≥8 (Utter et al., 2002).

### Physical Activity Training Intervention and Wait List Control Group

The physical activity intervention occurred for 2 h after each school day from September until May for 150 days of the 170-day school year. The program, Fitness Improves Thinking in Kids 2 (FITKids2; NICHD grant HD069381, www.clinicaltrials.gov, Identifier: NCT01619826) was based on the Child and Adolescent Trial for Cardiovascular Health (CATCH) curriculum (McKenzie et al., 1994) and aimed at improving aerobic fitness through engagement in a variety of developmentally appropriate physical activities. The environment was non-competitive and integrated activities such as fitness activities, motor skill practice, and organized games similar to tag (Castelli et al., 2011).

Within a daily lesson, children participated in moderate to vigorous physical activity (recorded by E600 Polar HR monitors; Polar Electro, Finland, and Accusplit Eagle 170 pedometers, San Jose, CA, USA) for 30–35 sustained minutes and then intermittently up to 90 min, thus exceeding the national physical activity guideline of 60+ minutes of moderate to vigorous physical activity per day (Centers for Disease Control and Prevention, 2012; U.S. Department of Health and Human Services, 2018). Overall, children spent ~50% of the time during the intervention engaged in moderate to vigorous physical activity (i.e., >70% of HR max, based on pre-test maximal HR from an incremental exercise test).

Each lesson began with the children completing stations that focused on a specific health-related fitness component (e.g., cardiorespiratory endurance, muscular strength). The activities were aerobically demanding and designed to encourage children to improve on previous performances by gradually increasing the number of repetitions or amount of resistance at a station. Although the stations were organized by health-related fitness components, each activity also required a motor or manipulative skill (e.g., dribbling a basketball around cones for 30-s, performing a sit-up, throwing a ball overhead). After the sustained participation and active rest rotations, the children consumed a healthy snack and were introduced to a themed educational component related to health promotion (e.g., goal setting, self-management). Each lesson concluded with the children participating in non-elimination, small group games, and activities such as dance or sports activities with modified rules selected from the CATCH curriculum. On the weekends, the children were encouraged to continue their participation in physical activity with their families, and physical activity worksheets were utilized during school holidays to log continued engagement. Average attendance across the 9-month intervention was 83.2% (*SD* = 14.12%).

The wait-list control group completed all facets of the baseline and post-intervention similar to those children who were randomized into the after-school physical activity program. As an incentive to stay in the study, children in the wait-list control group were allowed to participate in the physical activity program during the following school year (without involvement in any testing).

## Neuroimaging Methods

### Imaging Data Acquisition

T2*-weighted resting state images were acquired with a fast echo-planar imaging (EPI) sequence with blood-oxygen-level-dependent (BOLD) contrast [TA (acquisition time) = 4 min 6 s, TR = 2 s, TE = 25 ms, flip angle = 90°, 36 3.0 mm-thick slices acquired in ascending order, Grappa acceleration factor = 2, 92 × 92 matrix resolution, voxel size 2.6 × 2.6 × 3.0]. Participants were asked to lay still with eyes closed during the resting state scan.

To assist with registration, high-resolution structural MR scans were acquired using a 3D MPRAGE (Magnetization Prepared Rapid Gradient Echo) T1-weighted sequence with 0.9 mm isotropic resolution [TR = 1,900 ms; TE = 2.32 ms; TI = 900 ms (repetition/echo/inversion times)]. All images were collected on a Siemens Magnetom Trio 3T whole-body MRI scanner with a 12-channel receiver head coil (Siemens Medical Solutions; Erlangen, Germany).

### Imaging Data Analysis

#### Preprocessing

All imaging processing and analyses were carried out with a script library containing tools from FSL 5.0.4 (Functional Magnetic Resonance Imaging of the Brain’s Software Library^1^), AFNI^2^, FreeSurfer^3^, and MATLAB (The MathWorks, Natick, MA, USA; Voss et al., 2016; Weng et al., 2017).

For the resting-state fMRI data, a six-degree-of-freedom rigid-body head motion correction was applied to the fMRI data *via* AFNI’s 3dvolreg function, which produced six parameters of head motion (root-mean-squares of translational and rotational movement: X, Y, Z, pitch, roll, and yaw directions) for subsequent regression of spurious variance. Non-brain tissue was removed using BET, and spatially smoothing using a 6.0 mm three-dimensional Gaussian kernel of full-width at half-maximum was applied.

Then, after normalizing each global 4D dataset by the median intensity, we used an ICA-based method for further cleaning of motion-related artifacts (ICA-AROMA; Pruim et al., 2015). For baseline (pre-intervention) scans, ICA-AROMA yielded 28.5 ± 4.8 total independent components from the data, and it classified 16.7 ± 5.0 components as motion-related artifacts which were regressed out of the data (58.1 ± 12.8% of total components). ICA-AROMA removes motion-related variance from the BOLD data, and denoised volumes retain data from all time points.

Next, the denoised data were temporally filtered using AFNI’s 3dBandpass to ensure that the fMRI data fell within the frequency band of 0.008 < *f* < 0.08 Hz. This helps reduce unwanted noise such as high-frequency physiological signals (e.g., cardiac pulse) and low-frequency scanner drift. The frequency band was chosen to best represent the spontaneous, low-frequency fluctuation of the BOLD fMRI signal in the brain (Leopold et al., 2003; Salvador et al., 2005).

Following temporal filtering, the mean time series was extracted from three sources of non- neuronal variance: white matter signal from a region in white matter structure, the cerebrospinal fluid signal from a region in the lateral ventricle, and the global signal derived from a whole-brain mask. These nuisance signals were used as covariates to control for artifacts in the brain that may confound functional connectivity outcomes. With these three nuisance signals, the six head motion parameters obtained from the rigid body motion correction were band-passed with the same temporal filter applied to the fMRI data and included as nuisance regressors (Hallquist et al., 2013). Together, the nine band-passed nuisance regressors (white matter, CSF, global, and motion parameters) were entered into a multiple regression as independent variables predicting the resting-state fMRI data as a dependent variable using FSL’s FEAT tool.

Individual EPIs were registered to high-resolution structural T1 space using the boundary-based registration (BBR) algorithm (Greve and Fischl, 2009). First, high-resolution structural images were skull-stripped using FSL’s Brain Extraction Technique (BET) algorithm (Smith, 2002). Each skull-stripped anatomical image was visually inspected for errors. Then, registration of the EPIs from individual high-resolution structural space to standard MNI space was accomplished by FNIRT nonlinear registration with the default 10 mm warp resolution (Andersson et al., 2007a,b). The two resulting transformations were concatenated and then applied to the original functional image to create a functional image in standard MNI space; a reverse transform was used to register the seeds from standard MNI space to each participant’s native functional space.

#### Network Modularity Analysis

Our primary aim was to characterize modularity, a brain network measure that compares the number of connections within modules to the number of connections between modules. Modules were identified in a data-driven fashion using Newman’s spectral community detection (Newman, 2006). This approach identifies the optimal modular partition for each subject at each connection threshold.

For each participant, the preprocessed resting-state fMRI data was parcellated into 400 ROIs based on the Schaefer 2018 atlas (Schaefer et al., 2018). Then a 400 × 400 correlation matrix was generated by correlating the time-series between every possible pair of ROIs using Pearson’s coefficient and applying a Fisher z-transformation. Following previous reports, the resulting correlation matrices were thresholded and binarized over a range of connection density thresholds (2–10% at 2% increments; Power et al., 2011, 2012; Gallen et al., 2016; Baniqued et al., 2018, 2019). Modularity was calculated from unweighted and undirected brain graphs using the *modularity_und* tool from the Brain Connectivity Toolbox^4^. The middle 6% threshold was used for our primary analyses, and we verified the effects at the other thresholds.

### Statistical Analysis

A 2 (Group: intervention, wait-list) × 2 (Time: baseline, post-intervention) repeated measures analysis of variance (ANOVA) was conducted to explore the effect of time and the physical activity intervention on each cognitive outcome and aerobic fitness. Separate repeated-measures ANOVAs was conducted for each cognitive outcome. The repeated measures ANOVAs were conducted to confirm that cognitive performance improved from pre-intervention to post-intervention in our child sample, and to test whether the physical activity intervention had a greater effect on cognitive performance and aerobic fitness compared to the wait-list control group (a group of typically developing children over 9 months). Nevertheless, the main focus of the manuscript was to understand whether brain network modularity at baseline predicted intervention-related changes (improvements) in cognitive and scholastic performance.

Given our hypotheses, linear regressions were employed to test associations between brain modularity at baseline (pre-intervention) and change in cognitive performance and scholastic performance. Separate regressions were performed for children assigned to the physical activity intervention group and children assigned to the wait-list control group. Cognitive performance change scores were computed as the difference in post-intervention and pre-intervention (or baseline) scores for each participant. T-scores and standardized betas (β) are presented. The alpha level for all tests was set at *p* < 0.05. 95% confidence intervals (CI) were reported.

## Results

Brain network modularity at baseline was not significantly associated with age (*r* = −0.007, *p* = 0.93), sex (*r* = 0.004, *p* = 0.959), SES (*r* = 0.022, *p* = 0.79), IQ (*r* = −0.001, *p* = 0.991), pubertal timing (*r* = 0.027, *p* = 0.745), aerobic fitness (VO_2max_; *r* = 0.008, *p* = 0.922), or baseline performance for any cognitive outcomes (all *p* > 0.17).

### Changes in Aerobic Fitness, Cognitive Performance, and Scholastic Performance Across Time and Intervention

To begin, we explored the effects of time and the physical activity intervention on aerobic fitness and cognitive outcomes. There was no main effect of Time (*p* = 0.848) or Group × Time interaction (*p* = 0.961) for aerobic fitness.

There was a main effect of Time for the cognitive outcomes, with children in the physical activity group and wait-list control group showing improvements in cognitive and scholastic performance from pre-intervention (baseline) to post-intervention, as predicted (except for reading achievement; Table 1; Main effects of Time: Executive Processes: *F* = 36.441, *p* < 0.001; Cognitive Efficiency: *F* = 23.764, *p* < 0.001; Thinking Ability: *F* = 35.564, *p* < 0.001; Verbal Ability: *F* = 7.595, *p* = 0.007; Mathematics: *F* = 9.022, *p* = 0.003; Reading: *F* = 2.566, *p* = 0.111).

The Group (physical activity intervention, wait-list control) × Time (baseline, post-intervention) interaction did not reach significance for any of the cognitive outcomes, which suggests that the physical activity group did not show significantly greater gains in performance than the control group (Group × Time interactions: Executive Processes: *F* = 0.811, *p* = 0.369; Cognitive Efficiency: *F* = 1.969, *p* = 0.163; Thinking Ability: *F* = 0.319, *p* = 0.573; Verbal Ability: *F* = 1.340, *p* = 0.249; Mathematics: *F* = 0.136, *p* = 0.712; Reading: *F* = 2.211, *p* = 0.139).

Because of our *a priori* hypotheses predicting associations between baseline brain network modularity and gains in cognitive performance with an intervention, we explored associations between baseline brain network modularity and cognitive progress (change) by group.

### Baseline Modularity and Change in Cognitive Performance Clusters *via* Woodcock–Johnson

**Figure 2:**
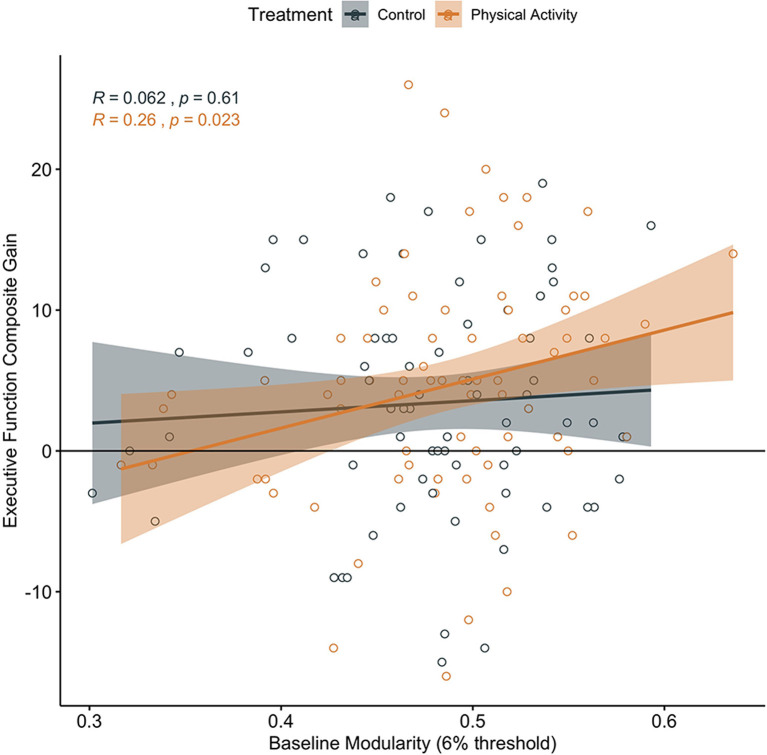
Association between baseline brain network modularity and change in executive function by group. Significant association in children involved in the physical activity intervention (uncorrected).

In children involved in the 9-month after-school physical activity intervention, higher brain network modularity at baseline was positively associated with a change in Executive Processes (*α* = 0.260, *t* = 2.328, *p* = 0.023, *N* = 77; CI: 0.0374366, 0.4817229; Figure 2).

**Figure 3:**
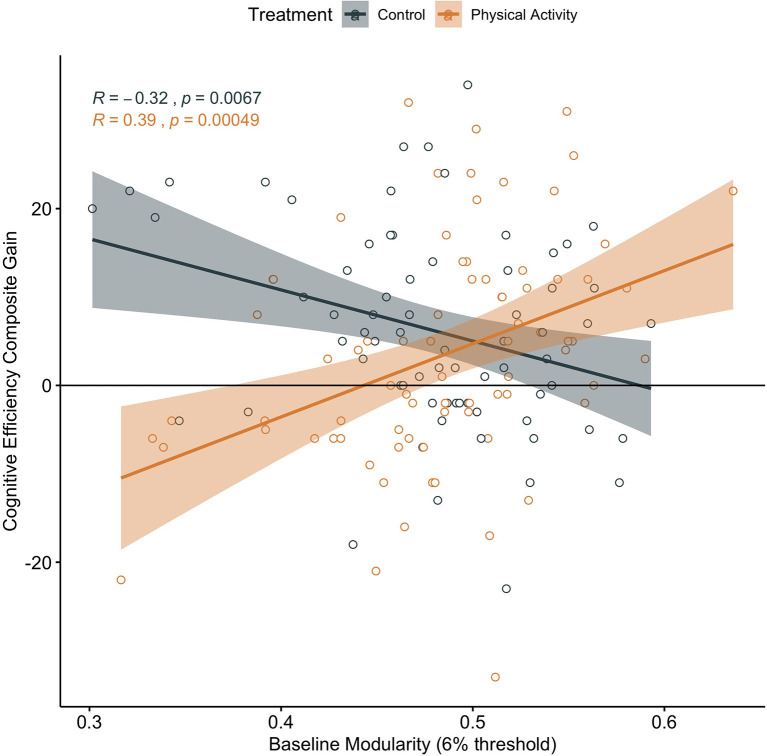
Association between baseline brain network modularity and change in cognitive efficiency by group. Significant association in children involved in the physical activity intervention (uncorrected and corrected).

In addition, in children in the physical activity group, higher brain network modularity at baseline was positively associated with a change in Cognitive Efficiency (*α* = 0.390, *t* = 3.647, *p* < 0.001, *N* = 76, CI: 0.1770542, 0.6035671; Figure 3). There were no significant associations between baseline modularity and change in Thinking Ability (*α* = 0.134, *t* = 1.160, *p* = 0.250, *N* = 76, CI: −0.09592174, 0.3631804) or change in Verbal Ability (*α* = −0.019, *t* = −0.161, *p* = 0.873, *N* = 76, CI: −0.2502647, 0.2129114).

Brain network modularity at pre-test did not positively predict cognitive performance changes in children in the wait-list control group (Executive Processes: *α* = 0.062, *t* = 0.520, *p* = 0.605, *N* = 71, CI: −0.1772713, 0.3021183; Figure 2; Cognitive Efficiency: *α* = −0.319, *t* = −2.794, *p* = 0.007, *N* = 71, CI: −0.5464083, −0.09114016; Figure 3; Thinking Ability: *α* = 0.066, *t* = 0.548, *p* = 0.585, *N* = 70, CI: −0.1750920, 0.3078135; Verbal Ability: *α* = 0.081, *t* = 0.672, *p* = 0.504, *N* = 71, CI: −0.1587927, 0.3199714). For Cognitive Efficiency in the wait-list control group, the effect was in the opposite direction than expected; that is, baseline brain network modularity negatively predicted change in performance, despite gains in performance from pre-intervention to post-intervention in the control group (Figure 3). Thus, this result was not in a meaningful direction for interpretation.

### Baseline Modularity and Change in Scholastic Performance

**Figure 4:**
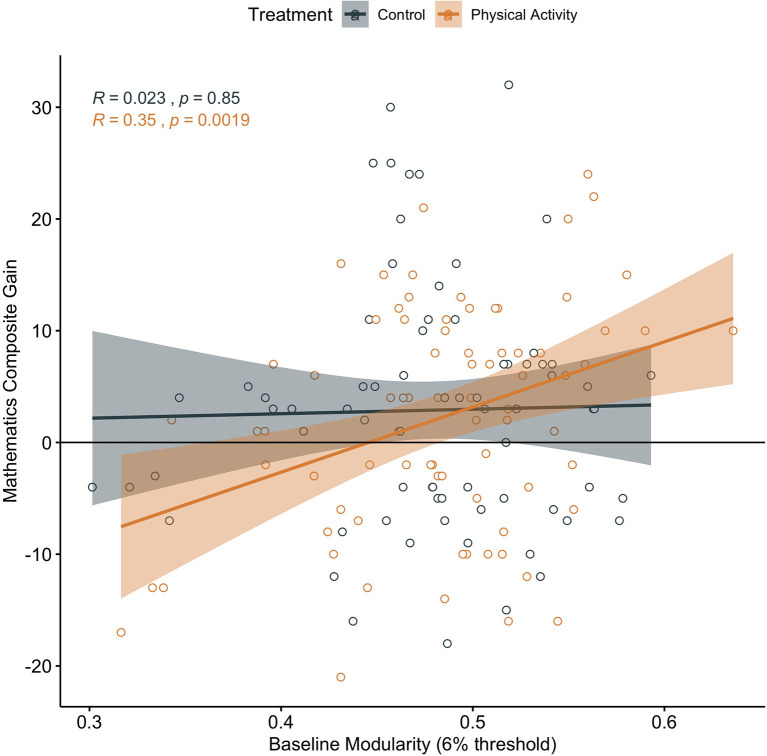
Association between baseline brain network modularity and change in mathematics achievement by group. Significant associations in children involved in physical activity intervention (uncorrected and corrected).

In children involved in the physical activity intervention, higher brain network modularity at baseline was positively associated with a change in mathematics achievement (*α* = 0.347, *t* = 3.221, *p* = 0.002; *N* = 78; CI: 0.1323028, 0.5609004; Figure 4). There was no significant association between baseline brain modularity and change in reading achievement (*α* = 0.001, *t* = 0.009, *p* = 0.993, *N* = 77, CI: −0.2290192, 0.2310368).

Brain network modularity at pre-test did not predict scholastic performance changes in children in the wait-list control group (Mathematics: *α* = 0.023, *t* = 0.193, *p* = 0.848, *N* = 72, CI: −0.2366569, 0.2366569; Figure 4; Reading *α* = 0.074, *t* = 0.618, *p* = 0.538, *N* = 72, CI: −0.1640388 0.3114265).

### Confirmation of Effects

We confirmed that the associations between brain network modularity at baseline and change in cognitive and scholastic performance remained significant in the physical activity group when controlling for age, sex, SES, IQ, pubertal timing, aerobic fitness, baseline performance, and in-scanner motion (mean of FD); Partial correlations between baseline modularity and change in performance for the children in the physical activity group: Executive Processes *r* = 0.276, *p* = 0.023; Cognitive Efficiency: *r* = 0.401, *p* = 0.001; Mathematics achievement: *r* = 0.379, *p* = 0.001.

### Bonferroni Correction

Note that when applying the Bonferroni correction for multiple comparisons (*p* = 0.05/6 tests; *p* = 0.0083), baseline modularity remained significantly associated with a change in Cognitive Efficiency and mathematics achievement in children involved in the physical activity intervention. Given the exploratory nature of our study which aimed to understand specific associations between brain network modularity and intervention-related changes in performance, we discussed all significant associations at both the Bonferroni-corrected and uncorrected levels.

## Discussion

Higher modularity of brain networks at baseline predicted greater improvements (changes) in cognitive performance (*via* cognitive performance clusters of executive function and cognitive efficiency) and scholastic performance (in particular, mathematics achievement) in children involved in an after-school physical activity intervention for 9 months. The relationships between baseline modularity and changes in performance were not present in a wait-list control group. The associations remained significant when accounting for age, sex, SES, IQ, pubertal timing, aerobic fitness, baseline performance, and in-scanner motion, which suggests that brain network modularity provides unique predictive information about intervention-related cognitive and academic progress during child development. Our results have important implications for biomarkers of cognitive plasticity in preadolescent children.

Our results generally support and extend previous research which demonstrates that brain network modularity at baseline predicts gains in cognitive performance in younger and older adults after cognitive and physical interventions (Arnemann et al., 2015; Gallen et al., 2016; Baniqued et al., 2018, 2019). Our extension of this research to children suggests that brain network modularity may predict cognitive changes *via* intervention in populations across the lifespan. We also extend the predictive power of brain modularity to other cognitive functions outside executive function, including the cognitive cluster of cognitive efficiency (which involves perceptual speed, short term memory, and the ability to store and recode information) as well as mathematics performance. Together, our data add to the framework of brain network modularity as a biomarker of plasticity and cognitive progress *via* interventions designed to improve cognitive and brain health (Gallen and D’Esposito, 2019). That is, global network properties and brain network architecture may capture individual differences in neuroplasticity that promote cognitive enhancement.

It is important to note that, unlike previous studies of modularity predicting intervention-related cognitive gains in young adults, older adults and TBI patients, relative to a control group (Arnemann et al., 2015; Gallen et al., 2016; Baniqued et al., 2018, 2019), physically active children in our study did not show significantly greater improvements in cognitive and scholastic performance compared to the wait-list control group, a group of typically developing children (age 7–9 years) across 9 months (i.e., lack of Group × Time interaction). That is, in our study, children in the physical activity intervention group and children in the wait-list control group showed statistically similar improvements in task performance across 9 months, perhaps due to practice effects and/or developmental effects that obscured potential benefits from the intervention. However, baseline modularity was only associated with changes in cognitive and academic performance in the physical activity group, not in the wait-list control group. As Gallen and D’Esposito (2019) suggest, modularity is a biomarker of intervention-related changes, so baseline modularity may have little predictive power for children not involved in a systematic multi-modal intervention. Indeed, the FITKids2 intervention was a multi-modal physical activity intervention, which included aerobically demanding activities as well as motor skills and health promotional activities. This intervention was different than the physical activity intervention in older adults, which involved walking around a track or dancing (Baniqued et al., 2018). Furthermore, the older adults in Baniqued et al. (2018) showed improvements in aerobic fitness levels with the physical activity intervention, unlike our study in which there were no significant effects of the physical activity intervention on aerobic fitness. As the physical activity dose provided in our intervention did not significantly modulate aerobic fitness levels, this may also help explain the lack of Group × Time interaction for all cognitive outcomes.

Computational models provide insight into the theoretical interpretations of the benefits of a modular network organization (Wig, 2017) as well as the association between modularity and neuroplasticity. For example, greater network modularity has been associated with better performance on memory tasks (Stevens et al., 2012; Chan et al., 2014), and brain modularity predicts rates of learning during working memory training (Iordan et al., 2018). Theoretically, individuals with a modular network organization may be able to apply small modifications and reconfigurations of specialized modules in response to new environments (e.g., interventions) to maximize performance (Kashtan and Alon, 2005). One research team compared functional connectivity during rest and during cognitive tasks to examine how changes in functional connectivity between rest and task contributed to cognitive performance (Schultz and Cole, 2016). Interestingly, instead of larger changes in functional connectivity reflecting optimization of networks during cognitive challenges, higher performers showed smaller changes in functional connectivity between rest and task, and network update efficiency correlated with intelligence (Schultz and Cole, 2016). These patterns suggest that small and efficient network updates may result in improved performance. As such, the results of the current study suggest that higher network modularity may represent an effective brain organization for predicting the progress of cognitive and academic performance with physical activity training during one school year.

More broadly, our results raise the possibility that brain network assessments in children may be used as biomarkers to guide the design and implementation of interventions to maximize effectiveness and improve outcomes. Metrics of modularity might be used to customize interventions, perhaps by personalizing intensity, frequency, and duration of physical activity for each child. For example, children with low baseline brain network modularity might require a longer or more vigorous physical activity intervention. Or, children with low baseline brain network modularity may not be at an optimal time point to benefit from an intervention. That is, it is possible that brain network modularity, to some degree, may signify a critical period of development when the developing brain is especially susceptible to intervention.

Future research is needed to determine how to maximize brain modularity at baseline to create optimal brain network properties to help individuals benefit from interventions. That is, what leads to increases in brain network modularity? It will also be important to understand the mechanisms by which brain modularity relates to changes in brain structure and function as well as neuronal health and vasculature with interventions. For example, in older adults, the upregulation of neurotrophic factors is associated with greater exercise-related changes in brain connectivity (Voss et al., 2013). Future studies might also include a longer resting-state scan–yet our scan (4 min, 6 s) was comparable to other brain modularity studies of varying resting-state scan length (4 min: Gallen et al., 2016; 5 min: Arnemann et al., 2015; 6 min: Baniqued et al., 2018, 2019). Furthermore, does brain network modularity predict changes in brain structure and brain function with interventions? Is the brain network modularity a biomarker for children with clinical disorders (e.g., Arnemann et al., 2015)? Do other network metrics such as global and local efficiency relate to changes in cognition? What subnetworks are contributing to the associations? For example, modularity in the association systems (e.g., default mode network, frontal-parietal network, dorsal attention network) has been shown to contribute to the association between modularity and intervention-related improvements in older adults (Gallen et al., 2016).

It will also be important to understand the specific cognitive processes predicted by modularity, as we do not report associations between baseline brain network modularity and changes in performance on tasks of thinking ability, verbal ability, or reading achievement. Indeed, our study was exploratory, as the first investigation to examine whether brain network modularity was a predictor of intervention-related changes in cognition and scholastic performance in children. As previous investigations have explored one cognitive outcome (executive function; Gallen et al., 2016; Baniqued et al., 2018, 2019) we aimed to understand whether modularity also predicted other cognitive abilities (e.g., cognitive efficiency, thinking ability), as well as school performance (e.g., mathematics, reading). Future studies should continue to dive into the specific associations between modularity and changes in intervention-related performance in populations across the lifespan as well as continue to consider multiple comparisons. Interestingly, the association between baseline modularity and change in executive function in children involved in the physical activity intervention was the one relationship that did not pass multiple comparison correction (unlike the significant associations between modularity and intervention-related changes in executive function in older adults; Gallen et al., 2016; Baniqued et al., 2018, 2019).

In conclusion, as trends indicate that children are becoming increasingly inactive and overweight, and physical activity opportunities are being reduced in schools, it is an important time to understand the associations among brain organization, cognitive and scholastic performance, and lifestyle factors such as physical activity. Interventions are designed for scientists and clinicians to better understand how to maximize neurodevelopmental processes important for cognitive performance and school achievement during childhood and across the lifespan. Given the time and cost to develop training interventions, it is important to develop biomarkers that predict how individuals respond to training as well as individual differences in cognitive and brain outcomes. It is of further importance to understand critical periods in the lifespan in which interventions may be especially effective. Here, we add to the evidence suggesting that brain network modularity, a measure of large-scale network organization, predicts change in cognitive function with an intervention, and we are the first to extend this framework to children.

## Data Availability Statement

The raw data supporting the conclusions of this article will be made available by the authors, without undue reservation.

## Ethics Statement

The studies involving human participants were reviewed and approved by Institutional Review Board, University of Illinois at Urbana-Champaign. Written informed consent to participate in this study was provided by the participants’ legal guardian/next of kin.

## Author Contributions

LC-H wrote the manuscript. LC-H, DC, CH, and AK conceived and designed the FITKids2 project and study. LC-H and TW analyzed the main outcomes of the present manuscript. LC-H, CK, ED, LR, S-CK, DW, and RW were involved in subject running and data organization of FITKids2 data. PB was included for experience with brain network modularity framework and feedback.

## Conflict of Interest

The authors declare that the research was conducted in the absence of any commercial or financial relationships that could be construed as a potential conflict of interest.

## Footnotes

^1^http://www.fmrib.ox.ac.uk/fsl

^2^http://afni.nimh.nih.gov/afni

^3^http://surfer.nmr.mgh.harvard.edu

^4^https://sites.google.com/site/bctnet/measures
